# Supplementary material for: Association between sites and severity of eczema and the onset of cow’s milk and egg allergy in children
Source: PLoS One. 2020 Oct 19;15(10):e0240980. doi: 10.1371/journal.pone.0240980 (PMC7571679; doi:10.1371/journal.pone.0240980)
Supplement: Attachment — Submitted filename: Response_to_Reviewers.docx [file pone.0240980.s002.docx]

*Reviewer #1: Major comments:*

*This article is a retrospective study on the association between CMA and EA and the sites and severity of infantile eczema. The study analyzed the data from patients aged 2 – 19 years with atopic disease and was treated in a pediatric allergy clinic. The data analysis has several limitations based on their experimental design, but the authors have clearly stated these limitations in their discussion. The data analyses support their conclusions that IgE-mediated CMA was positively correlated with severe facial eczema during infancy among children with atopic disease.*

*One major issue with the statistical analyses is that the author conducted multiple tests during their analyses. At the same time, they used a threshold P-value of 0.05 for significance, a typical cut-off when doing a single test. I would suggest that they use an adjusted p-value instead to control the false positive rate for this multiple test case.*

**RESPONSE:** We appreciate your suggestion. We have adjusted the p-values in Table 2 and 3 by Holm-Bonferroni correction and have added the following sentence in the Materials and Methods section; “The multiplicity of tests was adjusted by Holm-Bonferroni correction.” We have also changed the text accordingly.

*Minor comments:*

*Line 204: It is not very clear why the limitation would allow the authors to observe the relationship they mentioned. Maybe the authors could make the expression clearer for the readers.*

**RESPONSE:** We agree that the meaning of that sentence was not clear for readers. We have changed it to “However, we could observe specific relationship of eczema with CMA, but not with EA, in atopic children.”

*Reviewer #2: This manuscript describes a retrospective study about the relationship between eczema and cow milk or egg allergy in children. It demonstrated that the incidence of infantile eczema is higher in subjects with cow milk allergy, especially facial eczema. However, major concerns are also noticed as listed:*

1. *Language editing is recommended. Such as the usage of “whereas” in the Discussion section.*

**RESPONSE:** We appreciate your recommendation. We have asked the language review to the company again and attach the certificate of English editing.

2. *Does the paper consider any potential correlation between different sites of infantile eczema? The paper mentioned 289 subjects are involved in the study, while according to Table 2, there seems to be a few subjects with multiple sites of infantile eczema.*

**RESPONSE:** We have analyzed the correlation between different sites of eczema. The results are presented below:

Spearman’s correlation coefficient of each site of infantile eczema

| Eczema | Head | Face | Trunk | Upper limbs | Lower limbs |
| --- | --- | --- | --- | --- | --- |
| Head | 1 | 0.367  (P < 0.001) | 0.350  (P < 0.001) | 0.327  (P < 0.001) | 0.365  (P < 0.001) |
| Face | - | 1 | 0.358  (P < 0.001) | 0.381  (P < 0.001) | 0.377  (P < 0.001) |
| Trunk | - | - | 1 | 0.692  (P < 0.001) | 0.653  (P < 0.001) |
| Upper limbs | - | - | - | 1 | 0.834  (P < 0.001) |
| Lower limbs | - | - | - | - | 1 |

We have added the following sentences in the Materials and Methods and in the Results; “Spearman’s correlation coefficient was calculated for correlation between different sites of infantile eczema” and “Positive correlations were observed between all eczema site pairs. Strong correlations were found between the upper and lower limbs (correlation coefficient r = 0.83) and moderate correlations were found between the trunk and the upper limb (r = 0.69) and between the trunk and the lower limb (r = 0.65). The other pairs were weakly correlated (all r < 0.40).”

3*. In the Conclusion section, the paper states that the incidence of IgE-mediated CMA is higher in atopic children during their first year. However, in the “Material and methods” section, it says the collected data were from 2-19 years old patients. Meanwhile, I could not find any data mentioning any significant difference of CMA incidence during their first year.*

**RESPONSE:** We have mentioned “Caregivers of all patients were asked whether their children presented with eczema in the first year of life” in the Materials and Methods. However, for clearer expression, we have changed the prior sentence to “The study data were collected from electronic medical records and included infantile eczema, previous immediate allergic symptoms…”
